# Electronic Nose-Based Exhaled Volatile Organic Compound Pattern Recognition and Multivariate Signal Analysis for Discriminating Idiopathic Pulmonary Fibrosis from Autoimmune Usual Interstitial Pneumonia

**DOI:** 10.3390/s26092624

**Published:** 2026-04-23

**Authors:** Marcin Di Marco, Alessio Marinelli, Vitaliano Nicola Quaranta, Andrea Portacci, Esterina Boniello, Luciana Labate, Agnese Caringella, Anna Violante, Giovanna Elisiana Carpagnano, Silvano Dragonieri

**Affiliations:** Department of Traslational Biomedicine and Applied Neuroscience, Institute of Respiratory Disease, University “Aldo Moro”, 70121 Bari, Italy; marcin.dimarco@uniba.it (M.D.M.); alessio.marinelli@uniba.it (A.M.); vitalianonicola.quaranta@policlinico.ba.it (V.N.Q.); andrea.portacci@policlinico.ba.it (A.P.); agnese.caringella@policlinico.ba.it (A.C.); anna.violante@uniba.it (A.V.); elisiana.carpagnano@uniba.it (G.E.C.)

**Keywords:** enose, breathprint, VOCS, ILDs, IPF

## Abstract

Idiopathic pulmonary fibrosis (IPF) and autoimmune usual interstitial pneumonia (aUIP) share overlapping clinico-radiological features, complicating differential diagnosis. Electronic nose (eNose) technology characterizes exhaled breath profiles (“breathprints”) and may offer a non-invasive diagnostic approach in fibrotic interstitial lung diseases. To evaluate whether eNose breathprint analysis can discriminate between IPF and aUIP. In this cross-sectional study of 60 patients (34 IPF, 26 aUIP), breathprints were analyzed using principal component analysis (PCA, retaining eigenvalues > 1). Group differences were assessed via independent *t*-tests. Linear discriminant analysis (LDA) with leave-one-out cross-validation evaluated the discriminatory performance of PC combinations. PCA identified four principal components, with PC1 explaining 96% of the total variance. PC1 scores were significantly higher in aUIP compared to IPF (mean difference −0.53; 95% CI −1.04 to −0.02; *p* = 0.04); PC2-PC4 showed no significant differences (*p* > 0.3). LDA utilizing PC1 and PC3 achieved a cross-validated classification accuracy of 73.3% (95% CI 60.7–84.4, *p* < 0.05). eNose-derived breathprints showed preliminary discriminatory potential between IPF and autoimmune UIP, supporting further validation of this non-invasive adjunctive approach. Breathomics represents a promising non-invasive adjunctive tool for phenotyping fibrotic interstitial lung diseases, though larger validation studies integrating clinical and biological data are warranted.

## 1. Introduction

Interstitial lung diseases (ILDs) comprise a large and heterogeneous group of diffuse parenchymal lung disorders characterized by varying degrees of inflammation, fibrosis, and architectural distortion of the pulmonary interstitium. Although grouped under a common umbrella due to shared clinical and radiological manifestations, ILDs encompass entities with markedly different etiologies, pathogenetic mechanisms, prognostic trajectories, and therapeutic implications [1,2]. Among these, idiopathic pulmonary fibrosis (IPF) represents the archetypal progressive fibrosing ILD, characterized by relentless decline and poor prognosis [3]. In contrast, autoimmune-related interstitial pneumonias, frequently presenting with a usual interstitial pneumonia (UIP) pattern, arise secondary to connective tissue diseases and may follow a distinct biological course with different therapeutic responsiveness [4]. Differentiating IPF from autoimmune UIP (aUIP) remains one of the most challenging tasks in ILD diagnostics. Both conditions may exhibit nearly indistinguishable imaging features on high-resolution computed tomography (HRCT), including basal-predominant reticulation, traction bronchiectasis, and honeycombing [5,6]. Histopathological overlap further complicates interpretation, as the UIP pattern represents a morphological endpoint shared by multiple fibrosing processes rather than a disease-specific signature. Consequently, diagnosis relies heavily on multidisciplinary discussion integrating clinical, radiological, and pathological data [7]. While considered the diagnostic gold standard, this process is resource-intensive, time-consuming, and not universally accessible [7,8]. Current diagnostic tools present important limitations. HRCT may fail to provide a definitive pattern in a substantial proportion of cases, leading to classifications such as “probable UIP” or “indeterminate for UIP,” which perpetuate diagnostic uncertainty. Surgical lung biopsy can increase diagnostic confidence but carries significant procedural risks, including postoperative complications and non-negligible mortality, particularly in elderly or physiologically compromised patients [9,10,11]. Functional assessments such as pulmonary function testing and the six-minute walk test are indispensable for monitoring disease progression but lack sensitivity for early detection and provide limited specificity for etiological discrimination [5,12,13,14]. These constraints underscore a critical unmet need for non-invasive, repeatable biomarkers capable of capturing disease-specific biological information and supporting diagnostic stratification. Exhaled breath analysis, commonly referred to as breathomics, has emerged as a promising strategy for interrogating respiratory and systemic pathophysiology through the characterization of volatile organic compounds (VOCs) [15]. These compounds originate from endogenous metabolic pathways, oxidative stress reactions, inflammatory cascades, and interactions between host tissues and environmental exposures. The collective VOC profile, often termed the volatilome, constitutes a dynamic representation of ongoing biochemical processes and therefore offers the potential for real-time, non-invasive biomarker discovery. Unlike traditional biomarker strategies that focus on identifying single molecular entities, breathomics emphasizes pattern recognition across complex mixtures of VOCs. This shift reflects the understanding that multifactorial diseases such as ILDs are unlikely to be adequately described by isolated analytes but instead manifest as multidimensional metabolic signatures [16,17]. Indeed, prior investigations have demonstrated that analysis of composite VOC patterns yields greater discriminatory capacity than targeting individual compounds [18,19]. Breath analysis techniques can be broadly categorized into analytical chemistry platforms, such as gas chromatography–mass spectrometry (GC-MS), and pattern-recognition systems, most notably electronic nose (eNose) technologies. Analytical platforms enable precise molecular identification but require laboratory infrastructure, extended processing times, and specialized expertise. In contrast, eNose devices employ arrays of partially selective sensors that collectively respond to VOC mixtures, generating characteristic signal patterns that are interpretable through multivariate statistical or machine-learning algorithms. This sensor-based paradigm prioritizes classification performance, portability, and translational feasibility over molecular specificity, making it particularly attractive for point-of-care diagnostics. Originally developed for industrial applications such as environmental monitoring and food quality control, electronic noses have increasingly been adapted for biomedical use. Each sensor within an eNose exhibits partial sensitivity to a spectrum of VOCs rather than exclusive selectivity for a single compound. Diagnostic information emerges from the ensemble response of the sensor array, analogous to the combinatorial coding employed by the human olfactory system [20]. Recent developments in the field also include newer eNose architectures based on alternative sensing materials and miniaturized platforms, such as CNT-TiO2 hybrid nanostructure systems, further supporting the growing interest in sensor-array approaches for VOC discrimination across different biomedical and non-biomedical applications [21]. This architecture transforms disease detection into a signal-processing challenge. Raw sensor outputs generate high-dimensional datasets influenced by chemical composition, humidity, temperature, and flow dynamics. Extracting clinically meaningful information, therefore, requires rigorous preprocessing, dimensionality reduction, and supervised classification algorithms capable of identifying latent structures within complex data matrices. Multivariate statistical methods such as principal component analysis (PCA) and linear discriminant analysis (LDA) are particularly suited to this task, enabling transformation of correlated sensor responses into orthogonal features that maximize group separability [22,23]. From an engineering perspective, breathomics represents a systems-level measurement in which biological variability is not merely treated as noise but instead becomes part of the diagnostic signal. This conceptual framework aligns with the broader evolution toward data-driven phenotyping in precision medicine, where complex diseases are characterized through structured patterns rather than single biomarkers. A growing body of research has demonstrated that exhaled VOC profiles reflect key pathophysiological processes in ILDs, including oxidative injury, extracellular matrix turnover, and altered cellular metabolism [18,19]. Importantly, breathprint analysis has been shown to distinguish ILD patients from healthy individuals and from other respiratory diseases with high diagnostic accuracy, supporting its potential translational value [24,25,26,27]. Given the persistent diagnostic uncertainty surrounding UIP-pattern ILDs and the increasing maturity of sensor-based breath technologies, we hypothesized that multivariate analysis of eNose-derived breathprints could reveal discriminative metabolic signatures between IPF and autoimmune UIP. To test this hypothesis, we implemented a signal-processing workflow combining principal component analysis for dimensionality reduction with linear discriminant analysis for supervised classification. This approach enables the transformation of high-dimensional sensor outputs into interpretable latent variables while optimizing between-group separability. By integrating biomedical sensing, statistical modeling, and clinical phenotyping, the present study evaluates breathomics as a non-invasive adjunct for ILD characterization and contributes to the broader development of sensor-driven diagnostic strategies in respiratory medicine. We do not propose eNose analysis as a replacement for multidisciplinary discussion (MDD). Rather, this technology should currently be considered as a complementary, non-invasive adjunct that may help refine diagnostic suspicion in selected scenarios, such as early referral pathways or diagnostically challenging cases with overlapping fibrotic features after initial clinical and radiological evaluation.

## 2. Materials and Methods

### 2.1. Study Design and Population

This investigation was designed as a cross-sectional observational study aimed at evaluating whether exhaled breath patterns acquired through an electronic nose platform could discriminate between idiopathic pulmonary fibrosis (IPF) and autoimmune usual interstitial pneumonia (aUIP). A total of 60 consecutive patients were enrolled, including 34 diagnosed with IPF and 26 with autoimmune UIP. Diagnoses were established through multidisciplinary evaluation integrating clinical history, serological assessment, high-resolution computed tomography (HRCT), and, when available, histopathological findings, in accordance with international ILD diagnostic frameworks. Inclusion criteria were: age ≥ 18 years; confirmed diagnosis of IPF or autoimmune UIP; clinical stability at the time of breath sampling; and ability to perform controlled breathing maneuvers. To minimize confounding effects on volatile organic compound (VOC) composition, patients were excluded if they had an acute respiratory infection within 4 weeks prior to sampling, recent surgery or hospitalization, active malignancy, or inability to comply with breath collection procedures. These criteria align with recommendations emphasizing control of clinical variables known to influence breath biomarker composition [24,28,29].

### 2.2. Electronic Nose System Architecture

**Figure 1:**
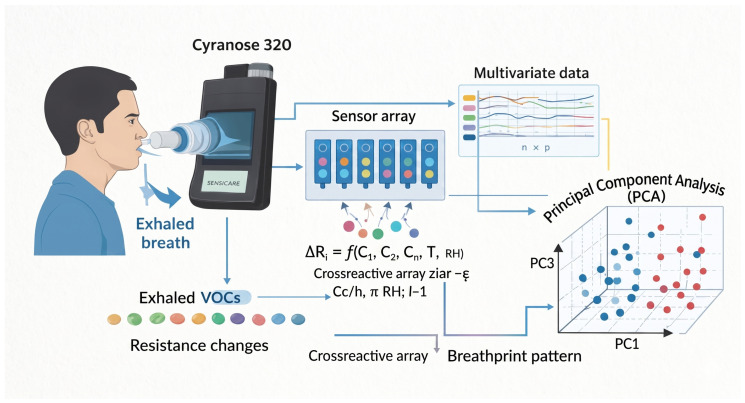
Schematic representation of the Cyranose 320 electronic nose operating principle for exhaled volatile organic compound (VOC) analysis.

Breath analysis was performed using a portable electronic nose (Cyranose 320, Sensigent, CA, USA), composed of a cross-reactive array of polymer-based chemical sensors (Figure 1). Unlike analytical platforms designed for molecular identification, the eNose functions as a pattern-recognition system in which classification is derived from the collective response of partially selective sensors exposed to complex VOC mixtures.

Each sensor undergoes a change in electrical resistance upon interaction with volatile compounds. The sensor response can be expressed as

(1) $$\Delta R_{i}=f\left(C_{1},C_{2},\ldots ,C_{n},T,RH\right)$$

where $C_{n}$ denotes the concentration of interacting VOC species, and *T* and RH represent ambient temperature and relative humidity, respectively. Because individual sensors respond simultaneously to multiple compounds, diagnostic information emerges from the multidimensional response vector rather than from any single sensor output. This combinatorial sensing principle mirrors biological olfaction and is particularly suited to detecting complex disease-related metabolic patterns.

### 2.3. Breath Sampling Protocol

Breath collection was standardized to reduce pre-analytical variability known to influence volatilomic measurements. Participants were instructed to refrain from eating, drinking (except water), or smoking for at least 2 h before testing and to avoid strenuous physical activity on the day of sampling. All measurements were performed using a disposable mouthpiece connected to the device. Environmental air sampling was conducted prior to each patient measurement to establish baseline VOC conditions. For controlled acquisition, subjects performed 5 min of acclimatized tidal breathing. Subsequently, a reproducible expiratory maneuver was used to deliver alveolar air into the sensor chamber. Specifically, patients were instructed to inhale to total lung capacity and exhale at a constant flow through the device until they reached residual volume.

Sensor exposure was maintained for a fixed acquisition interval, followed by a filtered-air purge phase to allow complete sensor recovery before the next measurement.

### 2.4. Signal Acquisition and Preprocessing

The eNose generated time-resolved resistance signals for each sensor, producing a multivariate temporal dataset. To compensate for environmental drift and baseline variability, sensor signals were normalized relative to pre-exposure resistance values according to:

(2) $$S_{norm}\left(t\right)=\frac{R\left(t\right)-R_{0}}{R_{0}}$$

where $R_{0}$ represents baseline resistance prior to exposure.

From each sensor response curve, characteristic features were extracted, including maximum amplitude, steady-state plateau value, response slope, and recovery dynamics. These descriptors summarize the interaction between the sensor array and the breath volatilome while reducing redundancy inherent to raw time-series data. The processed dataset was structured as

(3) $$X\in R^{n\times p}$$

where *n* = 60 subjects and *p* < 0.05, representing the total number of extracted features. Given the high dimensionality and expected collinearity among sensor features, dimensionality reduction was performed prior to classification.

### 2.5. Data Analysis

Principal component analysis (PCA) identified four principal components, with the first component (PC1) accounting for approximately 96% of the total variance. From a sensor physics perspective, this dominant component likely captures the large shared baseline signals inherent to human breath and environmental physics, rather than an isolated disease signature. As defined by our sensor response model, $\Delta R_{i}=f\left(C_{1},C_{2},\ldots ,C_{n},T,RH\right)$, the raw signal is heavily influenced by ambient temperature (T) and relative humidity (RH), alongside shared basal metabolic volatile organic compounds (VOCs) and inter-patient variations in expiratory flow dynamics. Furthermore, older-generation polymer composite sensors, such as those utilized in the Cyranose 320 platform, are known to exhibit baseline drift over time and marked sensitivity to moisture. Consequently, the diagnostically relevant, disease-specific volatilomic information differentiating IPF from autoimmune UIP is embedded within the lower-variance orthogonal components (e.g., PC3).

Mathematically, PCA decomposes the data matrix as

(4) $$X=TP^{T}$$

where *T* contains component scores, and *P* contains loadings. Components with eigenvalues greater than 1 were retained according to the Kaiser criterion. To evaluate diagnostic separability, linear discriminant analysis (LDA) was subsequently performed using combinations of retained principal components. LDA identifies a projection vector *w* that maximizes the variance between-classes while minimizing the variance within-classes:

(5) $$w=S_{W}^{-1}\left(\mu_{1}-\mu_{2}\right)$$

where $S_{W}$ represents the within-class scatter matrix and $\mu_{1},\mu_{2}$ denote class centroids. Model generalization performance was assessed using leave-one-out cross-validation (LOOCV). For each iteration, one subject was excluded from the training set, the model was trained on the remaining subjects, and the excluded subject was classified. This approach reduces optimistic bias in small datasets and provides a robust estimate of classification accuracy. All statistical analyses were performed using SPSS (IBM Corp., Armonk, NY, USA), version 31.0. A *p*-value < 0.05 was considered statistically significant.

## 3. Results

**Figure 2:**
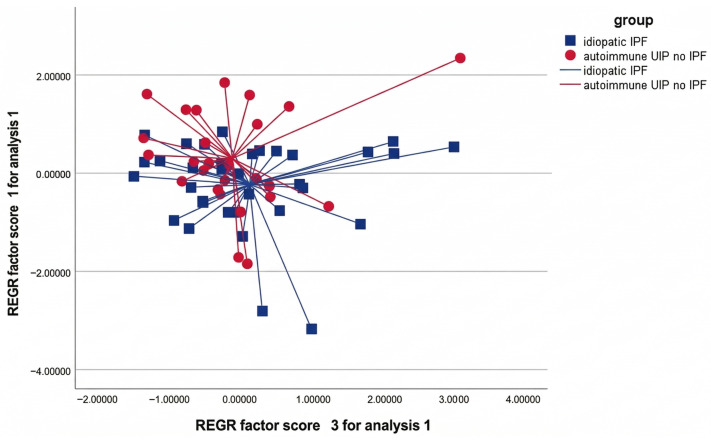
Discriminant plot of principal components (PC1 vs. PC3) in IPF and autoimmune UIP patterns. The two groups show a separation along PC1 and PC3 axes, consistent with the discriminant analysis indicating 73.3% cross-validated classification accuracy (*p* < 0.05). The PC1–PC3 projection is shown because this combination provided clearer class separation in the discriminant analysis than PC1–PC2, despite the lower proportion of variance explained by PC3.

A total of 60 patients were included in the final analysis, comprising 34 with idiopathic pulmonary fibrosis (IPF) and 26 with autoimmune usual interstitial pneumonia (aUIP). All participants successfully completed the breath acquisition protocol. No measurements were excluded due to technical failure, signal instability, or preprocessing artifacts, confirming the feasibility and robustness of the acquisition workflow. Principal component analysis (PCA) identified four principal components with eigenvalues greater than 1. The distribution of explained variance was markedly unbalanced: PC1 accounted for approximately 96% of total variance, whereas PC2–PC4 contributed only marginal additional variance. This pattern indicates the presence of a dominant shared signal component across the dataset. Comparison of PCA-derived scores between diagnostic groups revealed a statistically significant difference in PC1. Patients with aUIP exhibited higher PC1 scores compared with those with IPF, with a mean difference of −0.53 (95% CI −1.04 to −0.02; *p* = 0.04). In contrast, no statistically significant differences were observed for PC2, PC3, or PC4 (all *p* > 0.3) (Table 1). To evaluate classification performance, linear discriminant analysis (LDA) was applied using pairwise combinations of principal components. The optimal discriminative model was obtained using the combination of PC1 and PC3. This model achieved a cross-validated classification accuracy of 73.3% (95% CI 60.7–84.4), with statistical significance (*p* < 0.05) (Table 2 and Figure 2). Model stability was assessed using leave-one-out cross-validation. The classification behavior remained stable across iterations, with no evidence of systematic overfitting. Misclassifications were distributed across both diagnostic groups rather than concentrated within a single category, suggesting partial biological overlap between IPF and aUIP rather than model bias.

## 4. Discussion

In this study, we demonstrated that breathprint analysis obtained through an electronic nose platform, combined with multivariate statistical modeling, can identify measurable differences between idiopathic pulmonary fibrosis (IPF) and autoimmune usual interstitial pneumonia (aUIP). The classification model based on principal component-derived features achieved a cross-validated accuracy of 73.3%, indicating that exhaled volatilomic information contains disease-specific structure despite the shared UIP morphological pattern. These findings support the concept that fibrotic ILDs that appear radiologically and histologically similar may nevertheless differ at the metabolic level, and that such differences can be captured through pattern-recognition sensing. The UIP pattern represents a final common pathway of repeated epithelial injury, fibroblast activation, and extracellular matrix remodeling. However, the upstream biological drivers differ substantially between idiopathic fibrosis and autoimmune-mediated lung injury. Breathomics interrogates these upstream processes by detecting volatile metabolic by-products of oxidative stress, inflammation, and collagen turnover, thereby providing a systems-level readout of disease biology rather than structural change alone. This paradigm helps explain why no single volatile compound has proven sufficiently robust to serve as a standalone biomarker in ILD. Complex diseases generate distributed biochemical perturbations that are more effectively captured through multidimensional signal analysis than through isolated molecular measurements. Different approaches to exhaled breath analysis have been explored, each targeting distinct biological compartments. Exhaled breath condensate enables measurement of non-volatile mediators such as cytokines or hydrogen peroxide; however, its clinical applicability is limited by susceptibility to environmental and physiological confounders and by dilutional variability during sampling [30,31,32]. Similarly, exhaled nitric oxide provides insight into inflammatory processes but has demonstrated inconsistent discriminatory performance in ILDs and remains influenced by demographic and behavioral factors [33,34]. In contrast, VOC analysis—particularly when interpreted as an integrated “breathprint”—offers a dynamic representation of real-time metabolic activity. Rather than isolating individual molecules, this approach evaluates the collective volatilome, which more effectively reflects multifactorial disease mechanisms [16,17]. Electronic nose technologies operationalize this concept through cross-reactive sensor arrays coupled with multivariate modeling, transforming disease detection into a signal-classification problem rather than a molecular identification task. The diagnostic potential of breath analysis in fibrotic and inflammatory lung diseases has been increasingly explored over the past decade, with studies progressively shifting from single-compound identification toward multidimensional pattern-recognition strategies. This transition reflects the recognition that interstitial lung diseases are biologically complex and therefore better characterized through composite metabolic signatures. A substantial body of work has evaluated breath analysis technologies across diverse ILD populations using both electronic nose platforms and analytical chemistry-based volatilomic profiling. In a large multiclass study, Van der Sar et al. (2023) [29] evaluated patients with ILD alongside cohorts with asthma, COPD, and lung cancer using the SpiroNose device and partial least squares discriminant analysis (PLS-DA). The model demonstrated excellent discrimination of ILD from the other respiratory diseases, achieving an ROC-AUC of 0.99 in the test set. Earlier work by Dragonieri et al. (2020) [35], using the Cyranose 320 combined with PCA and linear discriminant analysis, showed that breathprints could distinguish IPF, COPD, and healthy controls with a cross-validated accuracy of 96.7% in an external validation cohort, with principal components correlating with bronchoalveolar lavage total cell count, suggesting a relationship between volatilomic signals and inflammatory burden. Krauss et al. (2019) [36] applied the Aeonose system to a heterogeneous ILD population and demonstrated strong discrimination between specific ILDs and healthy controls, whereas classification between fibrotic subtypes showed lower but still meaningful accuracy, reflecting biological overlap among ILDs. Using similar SpiroNose methodology, Van der Sar et al. (2022) [24] reported robust discrimination between sarcoidosis and healthy controls and good separation from other ILDs. A subsequent methodological comparison by the same group showed that feature selection combined with random-forest modeling improved classification performance, underscoring the importance of analytical strategy. In a smaller cohort of drug-induced ILD, breath analysis achieved moderate but significant discrimination versus healthy controls, with slightly improved performance in untreated patients [18,19]. Beyond fibrotic ILDs, electronic nose approaches have demonstrated discriminatory capacity in occupational lung disease, such as pneumoconiosis. Moor et al. (2021) [28] further confirmed near-perfect discrimination between ILD and healthy controls in a large cohort and maintained good performance when distinguishing IPF from non-IPF ILDs. Similarly, breath analysis has been shown to be capable of identifying systemic sclerosis-associated ILD independently of treatment status or disease severity [37]. Complementary analytical approaches have provided molecular-level confirmation of volatilomic alterations. Ion-mobility spectrometry, secondary electrospray ionization-mass spectrometry, and gas chromatography-based platforms have identified disease-specific VOC signatures and collagen-related metabolites in IPF, achieving diagnostic AUC values typically above 0.8 [18,38]. Importantly, several studies have demonstrated correlations between VOC profiles and functional indices such as total lung capacity, diffusing capacity, exercise performance, and even survival, suggesting that volatilomic signals capture dynamic pathophysiological processes rather than static phenotypes alone [39]. Taken together, these investigations demonstrate that breath-based technologies consistently detect biologically meaningful signatures across a spectrum of interstitial lung diseases. Diagnostic performance is strongest when distinguishing fundamentally different disease states and becomes inherently more nuanced when separating closely related fibrotic entities—an observation that mirrors the classification performance observed in the present study. The fact that discrimination was achieved through a multidimensional pattern-recognition approach rather than through a single dominant variable is consistent with the concept that breath signatures in fibrotic lung disease are complex and multifactorial. However, our results do not prove the biological multifactoriality of the signal; rather, they indicate that potentially relevant information was distributed across multiple correlated sensor-derived dimensions. The dominance of the first principal component in our dataset, accounting for the majority of variance, suggests the presence of a large shared metabolic background across fibrotic lung diseases. From a sensing perspective, this likely reflects common physiological processes such as basal metabolism, inflammation, and environmental exposure. Disease-specific information appears embedded within lower-variance components, explaining why combining principal components improved classification accuracy. This phenomenon is typical in complex sensor datasets, where diagnostically relevant information may reside in subtle variance structures rather than dominant signal axes. Breath composition is inherently influenced by numerous confounding variables, including sampling technique, expiratory flow dynamics, demographic characteristics, lifestyle factors, environmental exposures, medications, and comorbid conditions. These factors introduce analytical complexity but also underscore the integrative nature of breath as a biological matrix. Methodological heterogeneity across studies—from acquisition protocols to statistical modeling—remains a key barrier to clinical standardization. The current diagnostic gold standard for fibrotic interstitial lung diseases relies heavily on multidisciplinary discussion (MDD) integrating clinical, radiological, and pathological data. While highly accurate, this process is resource-intensive and not universally accessible. We do not propose exhaled breath analysis as a replacement for MDD. Rather, electronic nose technology represents a non-invasive adjunctive tool. Its clinical niche lies in supporting early diagnostic stratification, potentially acting as a triage instrument to prioritize high-risk patients for prompt MDD review, or serving as a supplementary modality to reduce diagnostic uncertainty in cases deemed ‘indeterminate for UIP’ following high-resolution computed tomography (HRCT), thereby potentially reducing the need for high-risk surgical lung biopsies. This study presents several notable limitations. Our linear discriminant analysis achieved a cross-validated classification accuracy of 73.3% using the combination of PC1 and PC3. While leave-one-out cross-validation (LOOCV) was applied to mitigate bias, this technique can exhibit high variance and may yield an optimistic estimate of model performance in relatively small cohorts. Therefore, these results must be interpreted as a preliminary proof-of-concept; external validation in a larger, independent, multicenter cohort is strictly required to confirm clinical generalizability. Additionally, while the sensor-based pattern-recognition approach prioritizes classification performance and portability, it inherently lacks molecular specificity. Future investigations must integrate electronic nose screening with analytical chemistry platforms, such as gas chromatography-mass spectrometry (GC-MS), to identify the specific biochemical compounds driving these latent structures and enhance our mechanistic understanding of the underlying fibrotic processes. An additional limitation relates to the use of the Cyranose 320, an earlier-generation eNose platform. As with other sensor-array systems, performance may be affected by sensor drift over time and by humidity sensitivity, both of which can influence signal stability and reproducibility. These technical constraints should be considered when interpreting exploratory findings and underline the need for validation with newer-generation devices and standardized acquisition workflows. Future research should prioritize protocol standardization, multicenter validation, and integration with advanced machine-learning approaches to improve robustness and reproducibility. Combining breathomics with imaging, functional, and molecular biomarkers may ultimately enable multimodal phenotyping strategies capable of capturing the full complexity of fibrotic lung disease. In this exploratory proof-of-concept study, eNose-based analysis of exhaled VOC patterns showed a moderate ability to discriminate idiopathic pulmonary fibrosis from autoimmune usual interstitial pneumonia. These findings support the potential role of breathprint analysis as a complementary, non-invasive diagnostic adjunct in fibrotic interstitial lung disease, but they remain preliminary and require confirmation in larger, externally validated cohorts.

## Figures and Tables

**Table 1:** Comparison of principal component scores between groups.

Principal Component	Idiopathic IPF (Mean ± SD)	Autoimmune UIP No IPF (Mean ± SD)	*p* Value
PC1	−0.23 ± 0.92	0.30 ± 1.04	0.04 *
PC2	0.02 ± 0.83	−0.02 ± 1.20	0.87
PC3	0.11 ± 1.08	−0.14 ± 0.89	0.35
PC4	−0.03 ± 0.98	0.04 ± 1.05	0.80

Note: Comparison of mean principal component (PC) scores between patients with idiopathic pulmonary fibrosis (IPF) and those with autoimmune usual interstitial pneumonia (UIP) without IPF. A significant difference was observed for PC1 (*p* = 0.04), while PC2–PC4 showed no significant variation between groups. Abbreviations: PC = principal component; IPF = idiopathic pulmonary fibrosis; UIP = usual interstitial pneumonia; SD = standard deviation.

**Table 2:** Discriminant analysis between IPF and autoimmune UIP patterns (cross-validated).

PC Combination	Cross-Validated Accuracy % (95% CI)
PC1 & PC2	55.0 (42.5–66.9)
PC1 & PC3	63.3 (60.7–84.4)
PC1 & PC4	53.3 (40.9–65.4)
PC2 & PC3	46.6 (34.6–59.1)
PC2 & PC4	51.7 (39.3–63.8)
PC3 & PC4	50.0 (37.7–62.3)

Note: Cross-validated classification accuracy using pairwise combinations of principal components (PCs) to discriminate idiopathic pulmonary fibrosis (IPF) from autoimmune usual interstitial pneumonia (UIP); 95% confidence intervals use Wilson binomial method with *n* = 60. Abbreviations: PC = principal component; IPF = idiopathic pulmonary fibrosis; UIP = usual interstitial pneumonia; CI = confidence interval.

## Data Availability

The original contributions presented in this study are included in the article. Further inquiries can be directed to the corresponding author.
